# Measured and perceived food environment influences on women’s nutritional health in marginalized areas of northern Mexico: a structural equation modeling analysis

**DOI:** 10.3389/fpubh.2025.1482256

**Published:** 2025-06-04

**Authors:** Zahid García, Juan Martín Preciado Rodríguez, Gloria Elena Portillo Abril, Alma Delia Contreras Paniagua, María Isabel Ortega-Vélez

**Affiliations:** ^1^Departament of Public Nutrition and Health, Centro de Investigación en Alimentación y Desarrollo (CIAD), Hermosillo, Sonora, Mexico; ^2^Department of Industrial Engineering, University of Sonora, Hermosillo, Sonora, Mexico

**Keywords:** food environment, structural equation modeling, obesity, health, marginalization, northern Mexico

## Abstract

**Background:**

Obesity is a growing global public health problem and a risk factor for developing non-communicable diseases (NCDs). The food environment is crucial in shaping nutritional behaviors and health outcomes. However, how food environment indicators interrelate and impact the population’s health in middle- and low-income countries is unclear. This study examined the association between the food environment and indicators of obesity and NCDs in adult women from medium and high-marginalization areas in Hermosillo, Northwest Mexico.

**Methods:**

A randomized sample of 104 adult women and 80 food retail stores participated in this cross-sectional study. Data on diet, anthropometric measurements, and NCD diagnoses were collected. We assessed the food environment’s personal (perceived) and external (measured) dimensions. Personal dimensions included perceived accessibility, affordability, convenience, and desirability of foods, while external dimensions comprised the variety, prices, density of food establishments, and advertising presence in participants’ neighborhoods. Data were collected via questionnaires and inventories and analyzed using geospatial and structural equation modeling (SEM) techniques to explore the relationships between food environment indicators and health outcomes.

**Results:**

The participants, with an average age of 47.6 years, exhibited an average BMI of 31.0 kg/m^2^, a high prevalence of abdominal obesity (90%), and NCDs (40%). The food environment in these areas was characterized by a high density and variety of food establishments offering unhealthy food options. Participants also perceived prices of healthy foods as high and reported exposure to advertising promoting unhealthy foods. Structural equation modeling revealed that a more nutritious food environment, as indicated by the perception of availability and lower prices of healthy foods, was negatively associated with waist circumference (*β*: −0.37, *p* < 0.05) and indirectly with the prevalence of NCDs (*β*: 0.30, *p* < 0.05).

**Conclusion:**

Our findings contribute to the empirical evidence that food environments influence the nutritional health of vulnerable populations. The results suggest that public policies should focus on improving the food environment by enhancing the availability and affordability of healthy foods.

## 1 Introduction

The food environment is the interface that mediates the interaction between individuals and food systems. It includes external dimensions such as food availability, prices, establishments, and advertising and personal dimensions like accessibility, affordability, convenience, and desirability. These dimensions are interrelated, with some preceding others (1). This definition and measurement of the food environment is being consolidated, especially in low- and middle-income countries. Researchers suggest the need to generate empirical evidence to test this theoretical proposal in these contexts (1, 2).

The food environment significantly impacts public health, shaping dietary patterns and nutritional health outcomes (3–5). Healthy food environments, characterized by the availability and affordability of nutritious foods, have been associated with improved diet quality and lower risks of obesity and NCDs (3, 6, 7). Conversely, specific indicators of unhealthy food environments, including the density of food stores selling ultra-processed foods high in calories, saturated fats, refined sugars, and sodium, contribute to unhealthy dietary patterns and an increase in the prevalence of obesity and NCDs (8–10).

Globally, NCDs, such as cardiovascular diseases, type II diabetes mellitus, and cancer, are leading causes of mortality, posing a significant public health challenge (11). Obesity and lifestyle (diet and physical activity) are key risk factors for developing these diseases (11). Public strategies to face these issues, have primarily focused on modifying individual behaviors (8, 12, 13). However, there is increasing recognition of the structural determinants of health, such as the availability of safe spaces for physical activity and the role of the food environment in shaping population health and nutrition (1, 11). For instance, in Mexico, rapid transformations in food retailing have adversely affected population health, particularly in marginalized areas where access to diverse and nutritious foods is limited (8, 14, 15).

Despite the growing evidence on the influence of the food environment on health and nutritional status, the interrelationship between the dimension of the food environment and outcome variables of interest is not clearly elucidated in diverse contexts (1, 16, 17). Furthermore, NCDs and obesity are complex phenomena that require holistic and multidimensional approaches (11, 18). In Hermosillo, Mexico, the marginalized areas with limited food establishment diversity experience adverse effects on food and nutritional security, underscoring the need for further investigation (14).

This cross-sectional study utilized data from adult women in medium- and high-marginalization areas of Hermosillo, Mexico, to assess the interrelationship of the personal (perceived) and external (measured) dimensions of healthy and unhealthy food environments with anthropometric indicators and NCDs. We hypothesize that unhealthy food environments are positively associated with higher anthropometric indicators and increased prevalence of NCDs, while healthy food environments exhibit the opposite trend.

## 2 Materials and methods

### 2.1 Study design and participants

**Figure 1:**
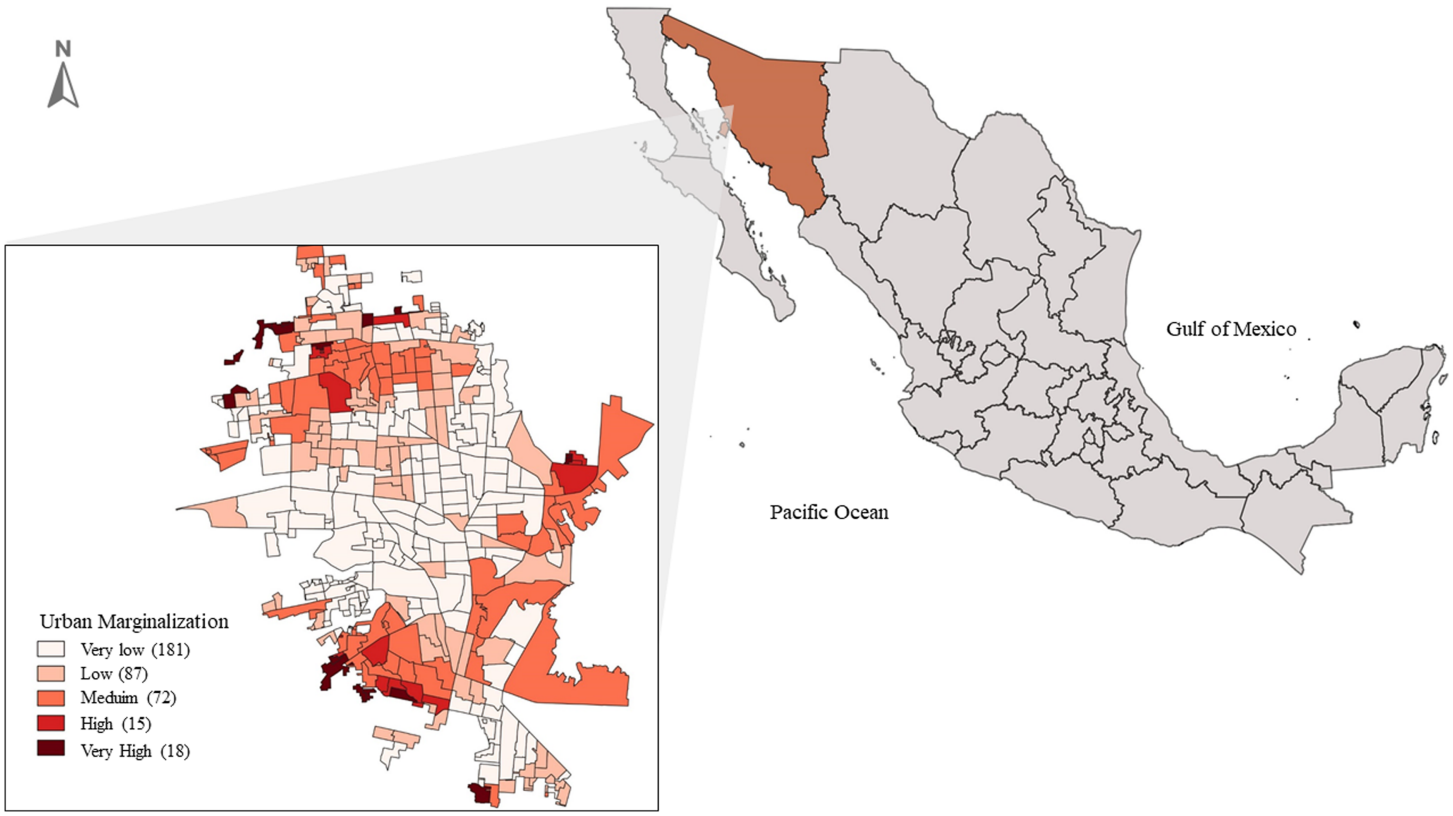
Map of AGEBs in Hermosillo, Sonora, Mexico, classified by urban marginalization. Source: INEGI (22). Hermosillo, Mexico, 2021.

This study uses an analytical cross-sectional design. The random sample included 104 adult women (18 years or older) and 80 food establishments, including supermarkets, corner stores, convenience stores, and fruit and vegetable stores located in Basic Geostatistical Areas (AGEBs) of medium and high marginalization in Hermosillo, Sonora (Figure 1). Hermosillo is the capital city of Sonora, a state located in the north Pacific of Mexico. It is located 270 kilometers south of the United States border and is the region’s key economic and administrative hub. Sonora’s gross domestic product (GDP) represents approximately 3.6% of Mexico’s national GDP, classifying it as a middle-income country. According to the 2020 Mexican census, Hermosillo has a population of over 855,000 inhabitants, and it is the largest city in Sonora and one of the most populated cities in northern Mexico. The AGEBs are geographic areas delineated by streets and avenues (19). Based on sociodemographic indicators such as educational level and literacy, housing characteristics, and household income, the National Population and Housing Council (CONAPO) classifies these areas into levels of urban marginalization, ranging from very low to very high (20). This study used AGEBs as a proxy for neighborhoods and as the spatial buffer defining the local food environment. We selected 24 out of the 87 AGEBs with medium and high marginalization in Hermosillo as the sampling frame, considering they had at least two food stores.

The sample size of 104 women was determined based on a power analysis using an effect size of *f*^2^ = 0.11, derived from previous studies on the association between food retail environments and obesity in the Mexican adult population (14). A statistical power of 80% and a significance level of *α* = 0.05 were considered, ensuring that the sample was adequate for assessing the proposed associations. According to Hair et al. (21), when using structural equation models, as in this study, the sample size should be at least 5 to 10 observations per estimated parameter. Additionally, sample sizes between 100 and 200 participants are advised (21). The inclusion criteria were women over 18 who could read, approve, and sign an informed consent form. Exclusion criteria included pregnant women or individuals with conditions that impaired their ability to complete the assessments. A two-stage random sampling method was applied to select participants. First, all households within the 24 selected AGEBs with medium and high marginalization levels were identified. Then, households were randomly selected in proportion to the total number of households within each AGEB, ensuring a balanced distribution. A woman who met the inclusion criteria was chosen in each selected household, and if no eligible women were present, the household was replaced.

The sample size for food establishments was determined using a 90% confidence level, maximum variability (*p* = 50%), and a 10% margin of error. This resulted in a sample size of 67 corner stores, corresponding to 20% of all such stores within the study AGEBs. The same 20% proportion was applied to other food establishment types, including supermarkets, convenience stores, and fruit and vegetable stores. Corner stores were the most densely distributed food establishments in the AGEBs included in the study. Therefore, they were used to calculate the sample size of food establishments. All establishments participating in this study were randomly selected. The Ethics Committee of the Research Center for Food and Development A.C. approved the study protocol (CEI/006/2021).

### 2.2 Individual variables

Participant information was collected between March and October 2021, when the federal government’s epidemiological traffic light system indicated a low risk of COVID-19 transmission. The team involved nutritionists trained and standardized by experienced personnel in the assessment instruments used in this study. Researchers adopted preventive measures such as social distancing, face masks, and disinfectants.

We used a questionnaire to collect sociodemographic variables, including age, length of residence, health services (no service, social security Mexican institute, public health institutions, and other private low-cost), occupation (housewife, employed, student), weekly income, educational level (elementary, high school, college), and general household characteristics (22). Socioeconomic status was determined using the Mexican Association of Market Intelligence and Opinion (AMAI) 8×7 guide, validated with the National Survey of Household Income and Expenditure (ENIGH) in Mexico (23). This questionnaire used household indicators such as monthly income, access to the internet, and bedroom numbers to classify socioeconomic status into low, medium, and high categories (23).

To assess the participants’ physical activity level, we used the International Physical Activity Questionnaire (IPAQ), a short version of the World Health Organization (WHO). This instrument aims to determine the type and amount of physical activity performed as part of daily life during the preceding 7 days of the interview (24). Participants were classified by activity level: low (mostly sedentary), moderate (activities like brisk walking or light chores on 3–5 days per week), and high (intense effort causing rapid breathing on at least 3 days per week) (24).

Diet information was obtained using a 24-h dietary recall following the multiple-pass method (25). This technique involves asking individuals about the foods and beverages consumed 24 h before the interview and recording them in a physical form. Subsequently, we organized the data in an Excel file and obtained food composition through dictionaries compiled from international, national, and regional databases (26–29). Additionally, foods were categorized based on national classification, including fruits, vegetables, grains and tubers, legumes, animal products, dairy, oils and fats, sugar-sweetened beverages, and ultra-processed foods, to determine the energy contribution of each food group (14, 30). The contribution of each food group was calculated by dividing the total energy (in kcal) provided by that group by the total energy intake (in kcal) from all foods consumed.

Participants’ weight, height, and waist circumference were measured by trained personnel, following standardized procedures (31, 32). We measured weight with a digital electronic scale (SECA 50–200 kg ± 0.05–0.1 kg), height with a portable stadiometer (SECA 2.1 m ± 1 mm), and waist circumference with a flexible measuring tape (± 1 mm). Body Mass Index (BMI) was calculated by dividing weight in kilograms by height squared in meters (kg/m^2^) and classified according to WHO standards: underweight <18.5, normal weight ≥ 18.5 and < 25, overweight ≥25 and < 30, obesity ≥30 (33). A waist circumference ≥ 80 cm, according to the International Diabetes Federation (IDF) criteria (34).

A pre-diagnostic questionnaire assessed the participant’s health status (presence of noncommunicable diseases) (30). In this questionnaire, participants reported whether they had previous diagnoses of diabetes, hypertension, or cardiovascular disease confirmed by a physician (30). We generated a dichotomous variable (Yes or No) to indicate whether participants had at least one noncommunicable disease.

### 2.3 Personal and external food environment

Table 1 describes the items used to assess the healthy and unhealthy personal food environment (perceived) domain. The validated food environment perception survey by Glanz and Green was the reference for the questionnaire (35). Two items assessed the availability perception of healthy (fruits, vegetables, fresh produce) and unhealthy foods (processed meats, snacks, candies, and sugar-sweetened beverages), two assessed the price perception, and one evaluated the perception of advertising for these foods. Additionally, one item assessed reasons for selecting food establishments. A Likert scale registered the responses ranging from 0 to 2. Since perceptions of food availability, prices, and advertising fluctuate depending on the type of establishment (grocery stores, convenience stores, supermarkets, and fruit and vegetable stores) frequented by participants, scores from Likert-scale responses for each kind of establishment were summed to obtain a continuous score.

**Table 1:** Items used to assess the healthy and unhealthy personal domain of the food environment.

Personal dimensions	Items	Likert scale response
Healthy food environment		
Perception of availability	Do you find fruits and vegetables available in the food establishments you frequent? Do you find fresh products available in the food establishments you frequent?	0 = No, 1 = Sometimes, 2 = Yes
Perception of prices	How do you consider the prices of fruits and vegetables in the food establishments you frequent? How do you consider the prices of fresh products in the food establishments you frequent?	0 = Cheap, 1 = Neither cheap nor expensive, 2 = Expensive
Perception of advertising	Do you observe advertisements encouraging you to buy healthy foods in the food establishments you frequent?	0 = No, 1 = Sometimes, 2 = Yes
Convenience	What is the main reason you choose the food establishments you frequent?	0 = Distance, 1 = Variety, 2 = Quality, 3 = Prices
Unhealthy food environment		
Perception of availability	Do you find sugary beverages, snacks, candies, and desserts available in the food establishments you frequent? Do you find processed meats available in the food establishments you frequent?	0 = No, 1 = Sometimes, 2 = Yes
Perception of prices	How do you consider the prices of sugary beverages, snacks, candies, and desserts in the food establishments you frequent? How do you consider the prices of processed meats in the food establishments you frequent?	0 = Cheap, 1 = Neither cheap nor expensive, 2 = Expensive
Perception of advertising	Do you observe advertisements encouraging you to buy unhealthy foods in the food establishments you frequent?	0 = No, 1 = Sometimes, 2 = Yes
Convenience	What is the main reason you choose the food establishments you frequent?	0 = Distance, 1 = Variety, 2 = Quality, 3 = Prices

Fresh products = Chicken, milk, meats, fish. Healthy foods = Fruits, vegetables, grains and tubers, legumes, water, unprocessed meats, dairy. Processed meats = Ham, chorizo, sausage. Unhealthy foods = Processed meats, sugary cereals, snacks and candies, fast food, sugary beverages. Hermosillo, Mexico, 2021.

The external (measured) food environment was assessed using four key indicators: (1) food variety, calculated as the average number of healthy and unhealthy food items available in each type of establishment; (2) food prices, collected through surveys with store managers to determine the cost of representative products in each food category; (3) advertising, quantified based on the presence of visible advertisements and promotions encouraging the purchase of healthy or unhealthy foods; and (4) food establishment density, calculated as the total number of establishments per square kilometer within each AGEB. Retail food establishments were categorized by the North American Industry Classification System (NAICS) according to the products and services they offer (36). Grocery stores include retail stores selling groceries, non-alcoholic beverages, and ice, which are small family-owned establishments offering ultra-processed and fresh foods (37). Fruit and vegetable stores, classified as retail stores selling fruits and vegetables, primarily provide fresh produce (38). Supermarkets, categorized as retail stores in supermarkets, include large commercial chains, such as Walmart, Ley (a regional chain), and Soriana (a national chain), offering a wide variety of fresh and processed foods (14, 37). Convenience stores, such as OXXO, Extra, 7-Eleven, and Circle K, operate 18 h a day, 365 days a year, specializing in ultra-processed beverages and foods (16). This study did not include retail food establishments selling exclusively fresh products, such as meat, poultry, and fish shops, dairy shops, or stores selling grains and seeds. This decision was based on previous studies with similar populations in the locality, emphasizing that participants typically acquire their food from grocery stores, supermarkets, fruit and vegetable stores, and convenience stores (9, 14).

Information about food retail stores in the AGEBs was obtained from the National Statistical Directory of Economic Units (DENUE), derived from economic censuses conducted by the National Institute of Statistics and Geography (INEGI), which contains data from formal establishments registered with the Secretary of Economy. Spatial distribution analysis of food retail stores was conducted using QGIS 3.16.3, georeferenced information from DENUE, and geographical delineations of AGEBs provided by INEGI. The density of establishments was calculated by dividing the number of food stores by the area (km^2^) of each AGEB.

### 2.4 Statistical analysis

Data analysis included IBM SPSS AMOS version 29.0 (SPSS, INC, Chicago Il., USA). We calculated means and standard deviations for continuous and discrete data and percentages for categorical variables. Analyses used Two-tailed tests with a significance level set at *p* < 0.05.

The healthy food environment was characterized by the availability and accessibility of fruits, vegetables, cereals and tubers, legumes, water, unprocessed meats, and dairy products in food establishments. The unhealthy food environment was defined by the presence of processed meats (ham, chorizo, sausages), sugary cereals, snacks, candies, fast food, and sugar-sweetened beverages (30). Healthy and unhealthy food environment indicators were analyzed and compared using the Wilcoxon non-parametric test for related data. A two-stage approach was employed for structural equation modeling to explore the interrelationship between healthy and unhealthy food environments with obesity indicators and the diagnosis of NCDs among participants.

In the first stage, we conducted two confirmatory factor analyses (CFA) to assess the fit of observed and latent (theoretical) variables. Model 1: Unhealthy food. The first model analyzed the unhealthy food environment and included two latent variables: personal domain environment and external domain environment. The personal domain environment was measured using four indicators: (1) perceived availability of unhealthy foods (perception score), (2) perceived price of unhealthy foods (perception score), (3) perceived advertising exposure for unhealthy foods (perception score), and (4) convenience (reason for selecting food stores). The external domain environment assessment included four indicators: (1) average variety of unhealthy foods in AGEBs, (2) average price of unhealthy foods in AGEBs, (3) average advertising of unhealthy foods in AGEBs, and (4) food store density.

Model 2: Healthy food. The second model analyzed the healthy food environment with the same two latent variables: personal domain environment and external domain environment. We measured the personal domain of the food environment by four indicators: (1) perceived availability of healthy foods (perception score), (2) perceived price of healthy foods (perception score), (3) perceived advertising exposure for healthy foods (perception score), and (4) convenience (reason for selecting food stores). The external domain assessment of the food environment involved four indicators: (1) average variety of healthy foods in AGEBs, (2) average price of healthy foods in AGEBs, (3) average advertising of healthy foods in AGEBs, and (4) food stores density.

In the second stage, structural equation models (SEM) were used to analyze the direct and indirect relationships between personal and external food environments with waist circumference and the presence of NCDs among participants. Waist circumference was used to indicate obesity due to its sensitivity in detecting abdominal obesity (higher cardio-metabolic risk) (38, 39). Models were adjusted for participants’ age (years) and energy intake (kcal).

Models were estimated using maximum likelihood estimation and evaluated with goodness-of-fit indices, including the chi-square test (χ2), comparative fit index (CFI), and root mean square error of approximation (RMSEA). An acceptable fit was considered for CFI values ≥0.9, and an RMSEA value less than 0.06 was deemed reasonable (40). Standardized regression coefficients and their respective significance values are presented in the models.

## 3 Results

### 3.1 Participant characteristics, diet and health outcomes

Table 2 describes the mean age of participants and years of residence in their neighborhood. The main socioeconomic level was medium, representing 65% of the participants. However, 22% lacked access to health services, representing a risk to their nutritional security. Additionally, although 80% of the participants engaged in moderate physical activity, most of these activities were related to household chores.

**Table 2:** General characteristics, anthropometric and participant’s health status.

Variable	*n* (x ± SD or Percentage)
General	
Age (years)	104 (47.6 ± 15.0)
Length of residence (years)	104 (20.3 ± 12.2)
Body mass index (BMI) (weight/height^2^)	104 (31.0 ± 5.70)
Waist circumference*	102 (100 ± 14.2)
Physical activity	
Low	15 (9.0%)
Moderate	21 (79.0%)
Intense	68 (12%)
BMI classification	
Normal	13 (12.5%)
Overweight	35 (33.7%)
Obesity	56 (53.8%)
Abdominal obesity*	
Yes	95 (94%)
Diabetes	
Yes	21 (20%)
Hypertension	
Yes	41 (40%)
Marginalization	
Medium	83 (80%)
High	21 (20%)
Education level*	
Elementary	23 (22.3%)
High School	35 (34%)
College	45 (43.7%)
Occupation	
Housewife	71 (68%)
Employed	29 (28%)
Student	4 (4%)
Health service	
No service	23 (22%)
IMSS	51 (49%)
Popular Service	15 (14%)
Others	11 (11%)
Socioeconomic status	
Low	32 (30%)
Medium	67 (65%)
High	05 (5%)

x ± SD: Mean ± Standard deviation; BMI, Body Mass Index; IMSS, Mexican Social Security Institute; *Variables with missing values. Hermosillo, Mexico, 2021.

**Figure 2:**
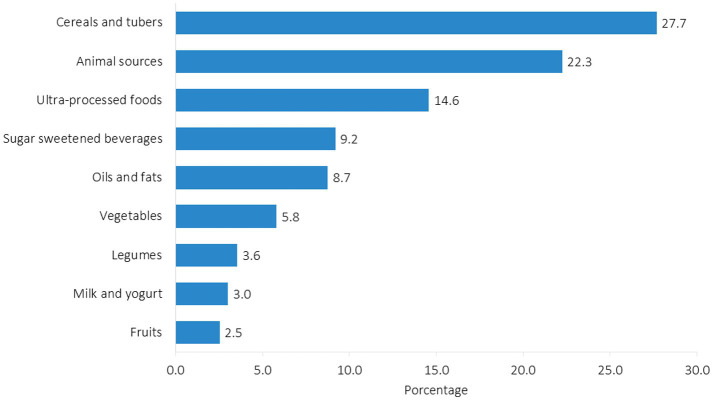
Energy contribution from different food groups consumed by participants (*n* = 104). Hermosillo, Mexico, 2021.

Dietary patterns indicated that cereals and tubers contributed the highest energy intake (74%), followed by animal source products and ultra-processed foods (those with added sugar and fat and sugar-sweetened beverages). The remaining 26% of energy came from oils, fats, vegetables, legumes, dairy, and fruits (Figure 2).

Anthropometric and health assessments showed a mean body mass index (BMI) of 31.0 ± 5.70 and an average waist circumference of 100 ± 14.2 cm. According to the WHO BMI classification, 88% of the participants were overweight and obese (41), and 90% had abdominal obesity. Furthermore, 20% of participants were diagnosed with diabetes and 40% with hypertension (Table 2).

### 3.2 The food environment and the participant’s health and nutritional status

Table 3 presents the general characteristics of the personal (perceived) and external (measured) domains of the healthy and unhealthy food environments. According to participant’s perceptions, prices for healthy foods are higher, and advertising for unhealthy foods is predominant in the food stores they usually attend (*p* < 0.05). Regarding external dimensions of the food environment, the average variety of foods, prices, and density of stores offering unhealthy foods are higher than healthy foods indicators (*p* < 0.05).

**Table 3:** General characteristics of the personal and external domains of healthy and unhealthy food environments.

Food environment dimensions	*n*	^a^Healthy foods		^b^Unhealthy foods		*p*
		*x*	SD	*x*	SD	
Personal dimensions						
Perception score of food availability	104	7.36	2.66	7.31	2.29	0.90
Perception score of food prices	104	4.58	2.14	3.78	2.25	0.01
Perception score of food advertising	104	1.40	1.50	2.32	1.75	0.01
External dimensions						
Average food variety in AGEBs	24	20.08	5.18	25.26	3.09	0.01
Average food prices in AGEBs	24	17.14	10.98	20.31	5.58	0.01
Average food advertising in AGEBs	24	11.77	16.38	18.21	33.85	0.35
^c^Food establishment density in AGEBs	24	2.51	2.44	31.77	14.67	0.01

^aHealthy foods = fruits, vegetables, cereals and tubers, legumes, water, unprocessed meats, and dairy products. bUnhealthy foods = processed meats, sweetened cereals, snacks and sweets, fast food, and sugary beverages. cSupermarkets and fruit and vegetables stores were grouped as establishments offering healthy foods, while convenience stores and grocery stores were grouped as establishments offering unhealthy foods. p = statistical significance in the Wilcoxon non-parametric test. Hermosillo, Mexico, 2021.^

**Figure 3:**
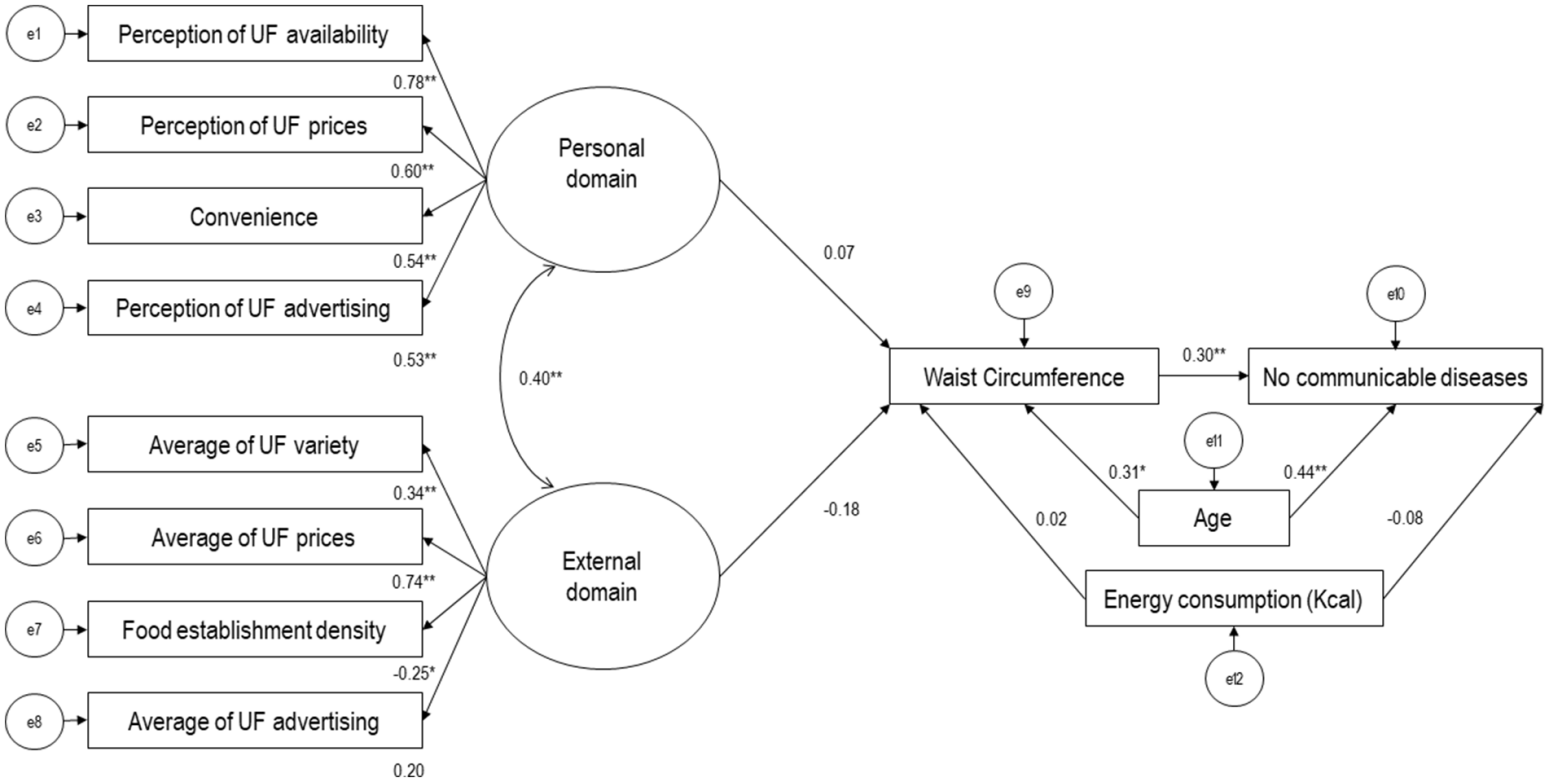
Model of the relationship between the unhealthy food environment, including observable variables (rectangles) and latent variables (ovals), waist circumference, and presence of noncommunicable diseases in participants (*n* = 104). Values are presented as standardized regression coefficients. UF = Unhealthy food (Processed meats, sugary cereals, snacks and candies, fast food, sugary beverages). e = error terms. *p < 0.05 **p < 0.001. Hermosillo, Mexico, 2021.

Figure 3 illustrates the interrelationship between the unhealthy food environment, waist circumference, and the presence of NCDs among participants. The latent variable (theoretical) “personal domain” was constructed from indicators including reasons for selecting food stores and participants’ perceptions of availability, prices, and advertising of unhealthy foods in their neighborhoods. The latent variable (theoretical) “external domain” included indicators such as total density of food stores, variety, prices, and average advertising of unhealthy foods in the study AGEBs. Among these indicators, those with the highest contribution (factor loading) to forming the latent variables were average prices of unhealthy foods and participants’ perceptions of the availability and prices of unhealthy foods (*β*: 0.74, *β*: 0.78, *β*: 0.60, *p* < 0.05, respectively). The latent variables of the unhealthy food environment, “personal domain” and “external domain” were positively correlated (*β*: 0.40, *p* < 0.05). After adjusting Model 1 for confounding variables (age and energy intake), there was no association between the personal and external latent variables of the unhealthy food environment and participants’ waist circumference (*β*: 0.07, *β*: −0.18, *p* > 0.05, respectively). However, on average, individuals with NCDs had a larger waist circumference (*β*: 0.30, *p* < 0.05). The structural equation model showed an acceptable fit (χ2/df = 1.44, *p* = 0.02, CFI = 0.90, RMSEA = 0.06).

**Figure 4:**
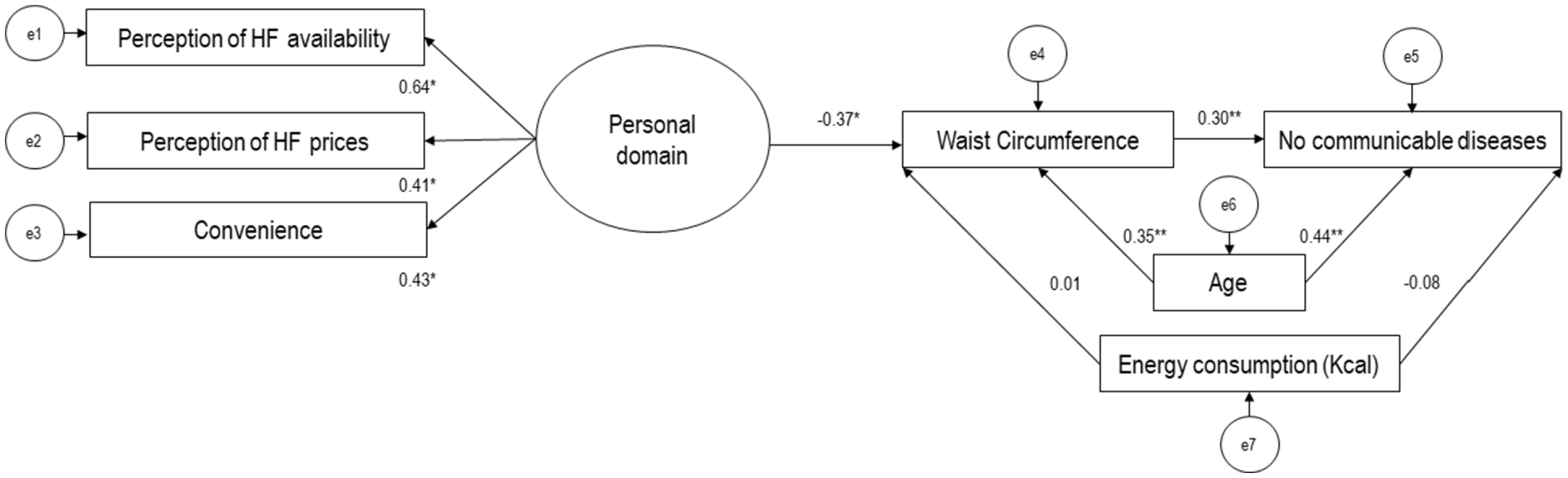
Model of the relationship between the healthy food environment, including observable variables (rectangles) and latent variables (ovals), waist circumference, and presence of noncommunicable diseases in participants (*n* = 104). Values are presented as standardized regression coefficients. HF = Healthy food (Fruits, vegetables, grains and tubers, legumes, water, unprocessed meats, dairy). e = error terms. *p < 0.05 **p < 0.001. Hermosillo, Mexico, 2021.

Figure 4 shows the interrelationship between the healthy food environment, waist circumference, and presence of NCDs among participants. In this model, the only latent variable (theoretical), “personal domain,” was constructed from indicators including reasons for selecting food establishments and participants’ perceptions of the availability and prices of healthy foods in their neighborhoods. The indicator with the highest factor loading for the latent variable was the participants’ perception of the availability of nutritious foods (*β*: 0.64, *p* < 0.05). The latent variable “external domain” was not included because its indicators (observable variables) did not significantly represent the theoretical construct.

After adjusting Model 2 for confounding variables (age and energy intake), the personal domain of the healthy food environment showed a negative association with participants’ waist circumference (*β*: −0.37, *p* < 0.05). Additionally, the personal food environment was indirectly associated with the presence of NCDs in participants through the mediation of waist circumference (*β*: 0.30, *p* < 0.05). However, there was no direct relationship between the personal food environment and the presence of NCDs. The structural equation model showed an acceptable fit (χ2/df = 0.84, *p* = 0.60, CFI = 0.95, RMSEA = 0.01).

## 4 Discussion

This study examined the interrelationship of the personal (perceived) and external (measured) domains of the food environment with obesity indicators and NCDs in a sample of adult women residing in marginalized areas of Hermosillo, Mexico. The findings provide empirical evidence on how food environments influence populations’ health.

According to the participant’s diet analysis results, cereals, animal source products, and ultra-processed foods were the food groups that contributed the most to energy intake. The latter include high energy content, saturated fats, refined sugars, sodium, and low fiber (39). This combination may be contributing to the high prevalence of obesity and NCDs observed among participants. Including ultra-processed foods in the diet modifies hormone levels that regulate satiety and increase insulin resistance and blood glucose levels (41). Therefore, these products are associated with weight gain and several health issues (40, 41). This diet pattern reflects the nutritional transition experienced by the Mexican population in recent years, driven by globalization, urbanization, and changes in food environments (42).

According to the general characteristics of the food environment, participants perceived healthy foods as expensive. Furthermore, their frequented establishments had high levels of advertising for unhealthy foods. Consistent with these findings, there was a greater variety and density of establishments offering unhealthy foods in the study’s AGEBs. These results highlight the need for public policies aimed at improving the availability and accessibility of healthy food options, as well as regulating the promotion of unhealthy foods.

Regarding the association between the food environment and participants’ obesity and health indicators assessed through structural equation models, the “personal “and “external” domains of the unhealthy food environment were significantly associated, which suggests that increased variety, prices, and advertising of unhealthy foods, along with decreased density of establishments in AGEBs, influence participants’ perceptions of availability, accessibility, convenience, and desirability of food in this study. These findings contribute to the empirical evidence supporting the theoretical model proposed by Turner et al., who emphasize that external dimensions of the food environment precede the internal dimensions and maintain a close relationship (1).

Nevertheless, the unhealthy food environment’s combined personal (perceived) and external (measured) domains were not associated with participants’ waist circumference. However, previous studies have found that unhealthy food environments are associated with a higher risk of obesity and NCDs (10, 15, 38, 43). Consistent with these studies, our analysis showed that participants perceived unhealthy foods as more affordable and heavily advertised, and there was a higher average variety of unhealthy foods and a higher density of establishments offering unhealthy foods. However, the lack of a significant direct relationship with waist circumference could be due to several reasons. First, the low variability in the availability and prices of unhealthy foods within the selected AGEBs may have limited the model’s ability to detect differences. Given the high density of stores offering ultra-processed foods in the study areas, exposure to an unhealthy food environment was relatively homogeneous, reducing the contrast necessary to identify significant relationships in a multivariate model (2). Second, prior studies have mostly relied on cross-sectional designs and bivariate associations, whereas our SEM approach accounts for complex interdependencies among multiple variables. This methodological difference could explain why a direct association was not observed. Additionally, the lack of significant findings does not necessarily imply the absence of an effect but rather suggests that other mediating or moderating factors, such as workplace food access or overall dietary patterns, may play a role in shaping health outcomes. Future research should explore these additional factors to provide a more comprehensive understanding of how the food environment interacts with individual-level behaviors (21). Additionally, the women in this study resided in areas of medium to high marginalization, which differs from scenarios in previous studies conducted in socioeconomically diverse settings.

On the other hand, the healthy food environment, consisting of a single latent variable, was directly associated with waist circumference and indirectly with NCDs among participants. This association suggests that better access to and perception of the availability of healthy foods may contribute to a lower risk of abdominal obesity. Furthermore, this healthy environment was indirectly associated with NCDs, mediated by waist circumference. This finding underscores the importance of promoting healthy food environments to prevent obesity and NCDs. A potential mechanism that could explain this relationship is that perceiving greater availability of healthy foods is closely related to a varied and healthy diet, which impacts individual nutritional health. Tani et al. (44) demonstrated that adults who perceived greater availability of fruits and vegetables in their neighborhood had lower mortality rates, attributed to better diet indicators.

From the perspective of Bronfenbrenner’s socio-ecological model (45), this study demonstrates that food systems, through the interaction of individuals with food environments, influence the nutritional status and health of the population. Our findings indicate that the food environment is a critical component in understanding why the border states and the Pacific-North region of the country have the highest rates of obesity in Mexico (30). In the context of the Sustainable Development Goals (SDGs), food systems play a crucial role in achieving those focused on combating various forms of malnutrition worldwide (46). However, our findings demonstrate that this goal must still be achieved, especially for vulnerable families. Therefore, it is imperative to drive changes toward agricultural and food systems, prioritizing health and sustainability at local, regional, and global levels (44).

Globally, strategies and public policy initiatives have been implemented to reduce the consumption of ultra-processed foods, obesity, and NCD, and could be adapted to the Mexican context. First, regulating unhealthy food advertising as seen in Chile, where marketing restrictions have reduced ultra-processed food consumption among children (47). Second, subsidies and incentives for healthy food sales could help increase access to fresh foods, following successful models from Brazil and the New Zealand, where incentives for fruit and vegetable producers and retailers have made nutritious options more affordable (6, 7). Third, zoning restrictions on convenience stores and fast-food outlets, a strategy implemented in some countries to reduce exposure to unhealthy food options near schools and hospitals, could be introduced in Mexico’s high-marginalization areas, where access to healthy foods is already constrained (48). Implementing these strategies could strengthen Mexico’s food policy framework and create healthier food environments, particularly in vulnerable areas, contributing to the long-term reduction of obesity and NCDs. In Mexico, for instance, front-of-package food labeling and taxes on sugar-sweetened beverages and ultra-processed products represent significant efforts to address public health issues (49, 50). However, these measures may not be sufficient, especially in marginalized population contexts. Additionally, public policies focused on modifying individual behavior appear insufficient in combating obesity and NCDs effectively. Comprehensive strategies that transform environments and address obesity through a complexity lens are required (51). Our findings contribute to the evidence of how social and structural factors, mediated through the food environment, can influence the development of obesity and NCDs. These findings align with the theory of complex systems, which advocates studying natural and social phenomena as interconnected systems rather than isolating their constituent parts (47, 52). In this context, the presence of NCDs and obesity among participants appears to be linked to indicators within the personal domain of the healthy food environment.

The strengths of this study are rooted in the empirical evidence generated around the theoretical model of the food environment proposed by Tuner and colleagues (6). This model categorizes and analyses various components of the food environment (personal and external domains), crucial for understanding how these factors influence dietary decisions and population health. Another strength was using structural equation models, a complex analysis that includes the interrelationships and interdependencies among multiple observable and latent variables. This approach provided insights into the impact of the food environment on health and nutrition from an integrative perspective. Additionally, the study employed a probabilistic selection of participants and food stores. However, it is essential to note that the findings are representative only of urban areas with medium and high levels of marginalization in the locality of Hermosillo, Mexico.

The results of this study must consider the following limitations: (1) Due to the cross-sectional design, only associations can be inferred and not causal relationships. Therefore, testing these models using longitudinal data in future studies is recommended. (2) The single 24-h dietary recall may not accurately capture habitual dietary intake due to day-to-day variability in food consumption. However, previous studies in similar populations have shown low intra-variability in diet (53, 54). (3) The prices of the most demanded foods were assessed by surveying establishment managers. However, this may only reflect the average prices of some foods available in those establishments. We attempted to cover various establishments to mitigate this limitation and used the most common prices reported. In future studies, employing more comprehensive pricing methods may be beneficial. (4) Data to calculate the density of retail food stores was obtained from DENUE, which records formal establishments. However, we realize that in Mexico and other middle- and low-income countries, there is an extensive variety of informal establishments offering foods and beverages. To address this limitation, we suggest future research to explore the impact of informal establishments on the food environment and public health, as proposed in previous studies (37). (5) Self-reported NCDs are a valid tool for epidemiological surveillance; nevertheless, this measure may underestimate diagnoses, for instance, the prevalence of diabetes in the Mexican population (55). (6) This study focused on the neighborhood food environment (AGEBs); nonetheless, other levels of the food environment, such as work, schools, and transportation routes, may influence individual outcome variables. Despite this limitation, the neighborhood environment substantially and directly impacts food choices and health. Still, future research would benefit from exploring how other environments contribute to health outcomes. (7) Obesity and NCDs are complex phenomena involving multiple factors not measured in this study, including genetic, biological, environmental, stress, and sleep factors (33). However, this study provides valuable insight into the relationships between the food environment and health. Future research should address these additional factors to understand the determinants of obesity and NCDs better.

## 5 Conclusion

The personal (perceived) food environment, defined by perceptions of availability, prices of healthy foods, and convenience, was directly associated with waist circumference and indirectly with NCDs in a sample of women residing in medium and high-marginalization areas in Hermosillo, Mexico. In this social, cultural, and socioeconomic context, and from a complex and interrelated perspective, through multivariate analysis, the evidence suggests that food systems, through food environments, significantly influence the health and nutritional status of vulnerable populations. These findings highlight food environments’ role in health outcomes and emphasize the need for public policies to focus on improving structural aspects, such as the food environment, instead of solely modifying individual behavior. Improving the availability and accessibility of healthy foods could significantly address public health challenges.

## Data Availability

The raw data supporting the conclusions of this article will be made available by the authors, without undue reservation.
